# Supplementary material for: Novel dual HIV maintenance therapy with nevirapine plus lamivudine retain viral suppression through 144 weeks—A proof-of-concept study
Source: PLoS One. 2020 Sep 23;15(9):e0237770. doi: 10.1371/journal.pone.0237770 (PMC7511013; doi:10.1371/journal.pone.0237770)
Supplement: S2 File — (PDF) [file pone.0237770.s002.pdf]

Kantonsspital St.Gallen

Klinik Infektiologie/Spitalhygiene

CH-9007 St.Gallen
Tel. 071 494 11 11
www.kssg.ch

NEVIRAPINE plus LAMIVUDINE (3TC) for HIV maintenance therapy – A single center pilot study

Clinical Study Protocol

NEVIRAPINE / 3TC PILOT

Study Type:	Investigator initiated, open label pilot study
Study Categorisation:	Risk category A
Study Registration:	Clinicaltrials.gov (planned)
Study Identifier:	BASEC Nr. 2016-01963 / EKOS 16/148
Sponsor-Investigator	Pietro Vernazza, Kantonsspital St. Gallen
Investigational Product:	Nevirapine and Lamivudine
Protocol Version and Date:	Version 1.3 – 12.4.2016<br>Amendment 12.4.17: Increase in sample size from 10 to 20

Signature Page(s)

Study number	BASEC Nr. 2016-01963 / EKOS 16/148
Study Title	NEVIRAPINE plus LAMIVUDINE (3TC) for HIV maintenance therapy – A single center pilot study

The Sponsor-Investigator and trial statistician have approved the protocol version V1.1 (dated 16.12.2016), and confirm hereby to conduct the study according to the protocol, current version of the World Medical Association Declaration of Helsinki, ICH-GCP guidelines or ISO 14155 norm if applicable and the local legally applicable requirements.

Sponsor-Investigator: Pietro Vernazza

Place/Date Signature

Local Principal Investigator at study site*:

I have read and understood this trial protocol and agree to conduct the trial as set out in this study protocol, the current version of the World Medical Association Declaration of Helsinki, ICH-GCP guidelines or ISO 14155 norm and the local legally applicable requirements.

Site	Klinik Infektiologie / Spitalhygiene, Kantonsspital St. Gallen
Principal investigator	Christian Kahlert

Place/Date Signature

Table of Contents

NEVIRAPINE / 3TC PILOT 1
STUDY SYNOPSIS 6
STUDY SUMMARY IN LOCAL LANGUAGE 8
ABBREVIATIONS 10
STUDY SCHEDULE 11
1. STUDY ADMINISTRATIVE STRUCTURE 12
1.1 Sponsor, Sponsor-Investigator 12
1.2 Principal Investigator(s) 12
1.3 Statistician ("Biostatistician") 12
1.4 Laboratory 12
1.5 Monitoring institution 12
1.6 Data Safety Monitoring Committee 12
1.7 Any other relevant Committee, Person, Organisation, Institution 12
2. ETHICAL AND REGULATORY ASPECTS 12
2.1 Study registration 12
2.2 Categorisation of study 12
2.3 Competent Ethics Committee (CEC) 12
2.4 Competent Authorities (CA) 13
2.5 Ethical Conduct of the Study 13
2.6 Declaration of interest 13
2.7 Patient Information and Informed Consent 13
2.8 Participant privacy and confidentiality 13
2.9 Early termination of the study 13
2.10 Protocol amendments 14
3. BACKGROUND AND RATIONALE 14
3.1 Background and Rationale 14
3.2 Investigational Product (treatment, device) and Indication 16
3.3 Preclinical Evidence 16
3.4 Clinical Evidence to Date 16
3.5 Dose Rationale / Medical Device: Rationale for the intended purpose in study (pre-market MD)16
3.6 Explanation for choice of comparator (or placebo) 16
3.7 Risks / Benefits 17
3.8 Justification of choice of study population 17
4. STUDY OBJECTIVES 17
4.1 Overall Objective 17
4.2 Primary Objective 17
4.3 Secondary Objectives 17
4.4 Safety Objectives 17
5. STUDY OUTCOMES 17
5.1 Primary Outcome 18
5.2 Secondary Outcomes 18
Other Outcomes of Interest 18
5.3 Safety Outcomes 18
6. STUDY DESIGN 18
6.1 General study design and justification of design 18

6.2 Methods of minimising bias 18
6.2.1 Randomisation 18
6.2.2 Blinding procedures 18
6.2.3 Other methods of minimising bias 18
6.3 Unblinding Procedures (Code break) 18
7. STUDY POPULATION 18
7.1 Eligibility criteria 18
7.1.1 Inclusion criteria 18
7.1.2 Exclusion criteria 19
7.2 Recruitment, screening, follow up 19
7.3 Criteria for withdrawal / discontinuation of participants 19
7.3.1 Definition of a rescue therapy 19
7.3.2 Procedures in case of viral failure 19
7.3.3 Premature study termination due to futility 19
8. STUDY INTERVENTION 19
8.1 Identity of Investigational Products (treatment / medical device) 19
8.1.1 Experimental Intervention (treatment / medical device) 19
8.1.2 Control Intervention (standard/routine/comparator treatment / medical device) 19
8.1.3 Packaging, Labelling and Supply (re-supply) 19
8.1.4 Storage Conditions 20
8.2 Administration of experimental and control interventions 20
8.2.1 Experimental Intervention 20
8.2.2 Control Intervention 20
8.3 Dose / Device modifications 20
8.4 Compliance with study intervention 20
8.5 Data Collection and Follow-up for withdrawn participants 20
8.6 Trial specific preventive measures 20
8.6.1 Procedures in case of viral failure 20
8.7 Concomitant Interventions (treatments) 20
8.8 Study Drug / Medical Device Accountability 20
8.9 Return or Destruction of Study Drug / Medical Device 20
9. STUDY ASSESSMENTS 20
9.1 Study flow chart(s) / table of study procedures and assessments 20
9.2 Assessments of outcomes 20
9.2.1 Assessment of primary outcome 20
9.2.2 Assessment of secondary outcomes 21
9.2.3 Assessment of other outcomes of interest 21
9.2.4 Assessment of safety outcomes 21
9.2.5 Assessments in participants who prematurely stop the study 21
9.3 Procedures at each visit 21
10. SAFETY 21
10.1 Reporting of serious adverse events (SAE) and other safety related events 21
11. STATISTICAL METHODS 21
11.1 Hypothesis 21
11.2 Determination of Sample Size 21
11.3 Statistical criteria of termination of trial 22

11.4 Planned Analyses 22
11.4.1 Datasets to be analysed, analysis populations 22
11.4.2 Primary Analysis 22
11.4.3 Secondary Analyses 22
11.4.4 Interim analyses 22
11.4.5 Safety analysis 22
11.4.6 Deviation(s) from the original statistical plan 22
11.5 Handling of missing data and drop-outs 22
12. QUALITY ASSURANCE AND CONTROL 22
12.1 Data handling and record keeping / archiving 22
12.1.1 Case Report Forms 22
12.1.2 Specification of source documents 23
12.1.3 Record keeping / archiving 23
12.2 Data management 23
12.2.1 Data Management System 23
12.2.2 Data security, access and back-up 23
12.2.3 Analysis and archiving 23
12.2.4 Electronic and central data validation 23
12.3 Monitoring 23
12.4 Audits and Inspections 23
12.5 Confidentiality, Data Protection 23
12.6 Storage of biological material and related health data 23
13. PUBLICATION AND DISSEMINATION POLICY 23
14. FUNDING AND SUPPORT 23
14.1 Funding 23
14.2 Other Support 23
15. INSURANCE 24
16. REFERENCES 24
17. APPENDICES 25

STUDY SYNOPSIS

Sponsor / Sponsor-Investigator	Pietro Vernazza, Kantonsspital St. Gallen
Study Title:	NEVIRAPINE plus LAMIVUDINE (3TC) for HIV maintenance therapy – A single center pilot study
Short Title / Study ID:	*Nevirapine/3TC Pilot* / BASEC Nr. 2016-01963 / EKOS 16/148
Protocol Version and Date:	Version *1.3*<br>*12/04/2016*
Trial registration:	Clinicaltrials.gov (intended)
Study category and Rationale	Open label pilot study. Principal goal is to demonstrate the efficacy of the intended maintenance regimen (NVP+3TC) as a basis for a large multicentre study within the Swiss HIV Cohort Study
Clinical Phase:	Phase 4 study – Pilot phase<br>Clinical Study Category A (see letter EKOS 12.12.16)
Background and Rationale:	Treatment of HIV infection requires life long medication. Reducing drug exposure has the potential to limit costs and side effects. Monotherapy with protease inhibitors or with integrase inhibitors have been conducted with reasonable short term (48wk) efficacy in approx. 80% of treated individuals. Combination of Nevirapine and 3TC has the potential of very limited adverse events and cost. Before testing this combination in a large comparative study, the feasibility of the approach should be demonstrated in a small pilot study.
Objective(s):	Demonstrate that Nevirapine/3TC mono-maintenance results in full viral load suppression for 6 months in at least 18 of 20 patients.
Outcome(s):	Viral load suppression defined as HIV-RNA < 50cp/ml over 24 weeks (with monthly measurements)
Study design:	Open label, single center, pilot study
Inclusion / Exclusion criteria:	Patients with no previous NNRTI failure and stable, fully suppressive long term HIV therapy (>6 months on Nevirapine, >24 months supressed)
Measurements and procedures:	Viral load measurement every 4 weeks up to 24 weeks
Study Product / Intervention:	Patient already on a stable Nevirapine treatment (with two nukes) will be continued with Nevirapine and lamivudine (3TC).
Control Intervention (if applicable):	n.a.
Number of Participants with Rationale:	20<br>The number is based on previous discussions with HIV experts in Switzerland regarding the requirement to convince them to initiate a large multicentre study within the SHCS. In a formal discussion of the protocol at the Scientific board of the SHCS, an extension to 20 patients was proposed
Study Duration:	24 week primary endpoint: full time until manuscript preparation: 12 months<br>The patients will have the option to continue therapy and observation will be continued in order to have more long-term information that can be used for the planned large multicentre trial.
Study Schedule:	Start of Inlusion: December 2016<br>Last patient reaching Wk 24: December 2017

Investigator(s):	Pietro Vernazza, Infektiologie, Kantonsspital St. Gallen<br>Christian Kahlert, Infektiologie, Kantonsspital St. Gallen
Study Centre(s):	Single center: Infektiologie, Kantonsspital St. Gallen
**Statistical Considerations:**	No formal sample size calculation possible. Other simplification-therapies (e.g. Dolutegravir Mono) have reported failure rates in the range of 20%. In previous discussions with other SHCS experts (e.g. Alexandra Calmy) experts would generally support the start of this nevirapine based multicentre study if a pilot study in at least 20 patients would reveal a success rate in 18 of 20 (90%) patients.
**GCP Statement:**	This study will be conducted in compliance with the protocol, the current version of the Declaration of Helsinki, the ICH-GCP or ISO EN 14155 (as far as applicable) as well as all national legal and regulatory requirements.

STUDY SUMMARY IN LOCAL LANGUAGE

Die HIV-Infektion wird standardmässig immer mit einer Kombination von drei HIV- Medikamenten behandelt. Der Grund für die Kombination mehrerer Substanzen ist vor allem das rasche Auftreten von Resistenzen, wenn nur mit einer oder zwei Substanzen behandelt wird.
In den letzten Jahren hat es sich jedoch gezeigt, dass die Gefahr einer Resistenzbildung deutlich geringer ist, wenn die Virusvermehrung im Körper einmal vollständig unterdrückt ist. Für diese sogenannte Therapie-Erhaltungsphase wurden nun zahlreiche Studien durchgeführt, welche versuchen, die Anzahl der Medikamente zu reduzieren.
Es gibt zahlreiche recht gute Erfahrungen mit Monotherapie. Einerseits mit Protease-Hemmern, neuerdings auch mit Integrase-Hemmern. Allerdings zeigen beide Gruppen eine Versagerrate der Monotherapie um ca. 10-20%. Man vermutet, dass die schlechte Penetration in den Genitaltrakt und ins Gehirn für dieses Resultat mitverantwortlich ist.
Im Gegensatz dazu zeigen neuere Studien zur Zweierkombination von Protease-oder Integrase-Hemmern mit 3TC eine sehr gute Wirksamkeit. Von 3TC wissen wir, dass es sehr gut im Gehirn und im Genitaltrakt wirkt.
Da die HIV-Infektion praktisch lebenslang eingenommen werden muss, ist eine Vereinfachung der Therapie von grosser Bedeutung. Sie reduziert nicht nur die potentiell immer möglichen Langzeit-Nebenwirkungen, sie reduziert auch Kosten.
Unser Zentrum hat ausgezeichnete Erfahrung mit einem bereits vor über 20 Jahren eingeführten HIV-Medikament, Nevirapine. Gut ein Drittel der Patienten ist mit dieser Substanz behandelt. Von den zusätzlichen Substanzen (Kivexa® oder Truvada®) enthalten beide 3TC (oder das analoge FTC) plus entweder Abacavir oder Tenofovir. Beide letztgenannten haben potentiell relevante Langzeit-Nebenwirkungen (Cardiovaskuläres Risiko resp. Nieren-/Knochen-Toxizität).
Eine Zweierkombination mit Nevirapine und 3TC hat erstens eine grosse Chance wirksam zu sein. Zweitens aber ist sie von allen alternativen Kombinationsmöglichkeiten zudem die günstigste. Nach allem was wir heute wissen, hat diese Kombination auch keine bekannten Langzeit-Nebenwirkungen.
Bei dieser Studie geht es um das prinzipielle „proof of concept“ um die vorgeschlagene Therapie in einer schweizweiten, durch den SNF finanzierten Studie zu prüfen.
Wir möchten mit dieser offenen, nicht kontrollierten Pilot-Studie erste Erfahrungen an 20 Patienten mit dieser Bi-Therapie sammeln, bevor wir ein grosses Studienprojekt einreichen.
Die Intervention besteht darin, dass die gut selektionierten Patienten (keine früheren Therapieversager auf NNRTI, schon längere Zeit stabile, gut supprimierende HIV Therapie mit Nevirapine) von ihrer Dreierkombination auf Nevirapine + 3TC umgestellt werden. Die Kontrolle der Wirksamkeit – der einzige Outcome-Parameter von Interesse – wird alle 4 Wochen erfolgen. Nach 24 Wochen ist die primäre Beobachtungsperiode abgeschlossen. Falls Patienten dies wünschen, können sie die Therapie fortsetzen. Wir würden den Verlauf prospektiv dokumentieren.
Alle Patienten werden laufend über allfällige Therapieversagen informiert, damit sie selbst entscheiden können, ob sie mit dem Studienmedikament weiter machen wollen. Zudem haben wir strikte Stopp-Kriterien definiert, aufgrund welcher die Studie wegen ungenügender Wirksamkeit abgebrochen würde.

Auch wenn wir schon gute Erfahrungen mit 3TC enthaltenden Bi-Therapien haben, so besteht auch bei dieser Studie ein Risiko eines Therapieversagens. Für jeden Patient wurde schon vor der Therapie bestimmt, welche „Rescue“ Therapie im Falle eines Therapieversagens eingesetzt werden könnte.

ABBREVIATIONS

AE	Adverse Event
CA	Competent Authority (e.g. Swissmedic)
CEC	Competent Ethics Committee
cART	Combination antiretroviral therapy
CRF	Case Report Form
ClinO	Ordinance on Clinical Trials in Human Research *(in German: KlinV, in French: OClin)*
eCRF	Electronic Case Report Form
CTCAE	Common terminology criteria for adverse events
DSUR	Development safety update report
GCP	Good Clinical Practice
IB	Investigator’s Brochure
Ho	Null hypothesis
H1	Alternative hypothesis
HFG	Humanforschungsgesetz (Law on human research)
HMG	Heilmittelgesetz
HRA	Federal Act on Research involving Human Beings
IMP	Investigational Medicinal Product
IIT	Investigator-initiated Trial
ISO	International Organisation for Standardisation
ITT	Intention to treat
KlinV	Verordnung über klinische Versuche in der Humanforschung *(in English: ClinO, in French OClin)*
LPTh	Loi sur les produits thérapeutiques
LRH	Loi fédérale relative à la recherche sur l’être humain
MD	Medical Device
NNRTI	Non-nucleoside RT-Inhibitor
OClin	Ordonnance sur les essais cliniques dans le cadre de la recherche sur l'être humain *(in German : KlinV, in English : ClinO)*
PI	Principal Investigator
SDV	Source Data Verification
SHCS	Swiss HIV Cohort Study
SOP	Standard Operating Procedure
SPC	Summary of product characteristics
SUSAR	Suspected Unexpected Serious Adverse Reaction
TMF	Trial Master File

STUDY SCHEDULE

Study Periods	Screening	Primary observational phase			Follow up for the time patient remains on 3TC+NVP
Visit	1	2	3 to 7	8	
Time (week)	0 to - 4	0	4 (+/-5d), 8, 12, 16, 20 (all +/- 10d)	24 +/-1 wk	Every 12 wks (up to wk 96)
Patient Information and Informed Consent	X				
Demographics	X				
HIV-treatment history	X				
In- /Exclusion Criteria	X				
Definition of rescue therapy	X				
Vital Signs	X			X	X
HIV-RNA	X		X	X	X
Concomitant Therapy		X	X	X	X
Adverse Events			X	X	X

1. STUDY ADMINISTRATIVE STRUCTURE

n.a.

1.1 Sponsor, Sponsor-Investigator

Prof. Dr. med. Pietro Vernazza (Sponsor – Investigator)

Klinik Infektiologie Spitalhygiene, Kantonsspital, 9007 St. Gallen, +41 71 494 2631

1.2 Principal Investigator(s)

Prof. Dr. med. Pietro Vernazza (Sponsor – Investigator)

Klinik Infektiologie Spitalhygiene, Kantonsspital, 9007 St. Gallen, +41 71 494 2631

Dr. med. Christian Kahlert

Klinik Infektiologie Spitalhygiene, Kantonsspital, 9007 St. Gallen, +41 71 494 1971

1.3 Statistician ("Biostatistician")

None

1.4 Laboratory

ZLM, St. Gallen, Switzerland (Kantonsspital, 9007 St. Gallen)

1.5 Monitoring institution

n.a.

1.6 Data Safety Monitoring Committee

n.a.

1.7 Any other relevant Committee, Person, Organisation, Institution

None

2. ETHICAL AND REGULATORY ASPECTS

Before the study will be conducted, the protocol, the proposed patient information and consent form as well as other study-specific documents shall be submitted to a properly constituted Competent Ethics Committee (CEC) and/or competent authorities.

The clinical study can only begin once approval from all required authorities has been received. Any additional requirements imposed by the authorities shall be implemented.

2.1 Study registration

Study registration at clinicaltrials.gov has been performed (Dec. 15, 2016). Review is pending.

2.2 Categorisation of study

Risk Category A. See decision of CEC dated 12.12.2016

2.3 Competent Ethics Committee (CEC)

Approval of the EKOS (CEC: Ethikkommission Ostschweiz) is a mandatory condition before the study is started.

Any change of the protocol or in the conduct of the study, any unanticipated problem or premature study termination as well as the final report will be reported to the CEC.

Premature study end or interruption of the study is reported within 15 days. The regular end of the study is reported to the CEC within 90 days, the final study report shall be submitted within one year after study end. Amendments are reported according to chapter 2.10.

2.4 Competent Authorities (CA)

With risk category A, approval by Swissmedic is not required

2.5 Ethical Conduct of the Study

The study will be carried out in accordance to the protocol and with principles enunciated in the current version of the Declaration of Helsinki, the guidelines of Good Clinical Practice (GCP) issued by ICH, in case of medical device: the European Directive on medical devices 93/42/EEC and the ISO Norm 14155 and ISO 14971, the Swiss Law and Swiss regulatory authority's requirements. The CEC and regulatory authorities will receive annual safety and interim reports and be informed about study stop/end in agreement with local requirements.

2.6 Declaration of interest

None of the investigators has a conflict of interest related to this study.

2.7 Patient Information and Informed Consent

The investigators will explain to each participant the nature of the study, its purpose, the procedures involved, the expected duration, the potential risks and benefits and any discomfort it may entail. Each participant will be informed that the participation in the study is voluntary and that he/she may withdraw from the study at any time and that withdrawal of consent will not affect his/her subsequent medical assistance and treatment.

The participant must be informed that his/her medical records may be examined by authorised individuals other than their treating physician.

All participants for the study will be provided a participant information sheet and a consent form describing the study and providing sufficient information for participant to make an informed decision about their participation in the study. Participants will receive the patient information sheet at least one day in advance.

All participating patients will be informed in writing or by phone (within 48 hrs) call about any single (confirmed) treatment failure having occurred within the study. Patients who miss a follow-up will be contacted by phone and/or mail. Rigorous effort will be made in order to clarify reasons for lost to follow-up each study participant.

The patient information sheet and the consent form will be submitted to the CEC and to the competent authority (as applicable) to be reviewed and approved. The formal consent of a participant, using the approved consent form, must be obtained before the participant is submitted to any study procedure.

The participant should read and consider the statement before signing and dating the informed consent form, and should be given a copy of the signed document. The consent form must also be signed and dated by the investigator (or his designee) and it will be retained as part of the study records.

2.8 Participant privacy and confidentiality

The investigator affirms and upholds the principle of the participant's right to privacy and that they shall comply with applicable privacy laws. Especially, anonymity of the participants shall be guaranteed when presenting the data at scientific meetings or publishing them in scientific journals.

Individual subject medical information obtained as a result of this study is considered confidential and disclosure to third parties is prohibited. Subject confidentiality will be further ensured by utilising subject identification code numbers to correspond to treatment data in the computer files.

For data verification purposes, authorised representatives of the Sponsor (-Investigator), an ethics committee may require direct access to parts of the medical records relevant to the study, including participants' medical history.

2.9 Early termination of the study

The Sponsor-Investigator may terminate the study prematurely according to certain circumstances, for example:

- ethical concerns,
- insufficient participant recruitment,
- when the safety of the participants is doubtful or at risk, respectively (see study termination

rule)

- alterations in accepted clinical practice that make the continuation of a clinical trial unwise,
- early evidence of benefit or harm of the experimental intervention

2.10 Protocol amendments

Substantial amendments are only implemented after approval of the CEC and CA respectively.

Under emergency circumstances, deviations from the protocol to protect the rights, safety and well-being of human subjects may proceed without prior approval of the sponsor and the CEC/CA. Such deviations shall be documented and reported to the sponsor and the CEC/CA as soon as possible.

All Non-substantial amendments are communicated to the CA as soon as possible if applicable and to the CEC within the Annual Safety Report (ASR).

3. BACKGROUND AND RATIONALE

3.1 Background and Rationale

Since 1996, when combined antiretroviral treatment of HIV (cART) with three antiretroviral compounds was established, complete and continued suppression of HIV replication within the body could be achieved. This status of viral control leads to an impressive resolution of most HIV-associated condition and almost normalizes the immune-activation associated with HIV-infection(1). Once suppression of viral replication is stably reached, no further viral replication and evolution occurs in the body as long as uninterrupted treatment is maintained(2). As a result, life expectancy of HIV infected individuals has almost reached the levels of the general population(3).

Until today, all International guidelines discuss the use of certain antiretroviral combinations to start cART(4). Once treatment is established, HIV infected individuals usually continue with their therapy unless the occurrence of side effects require a change of one or more compounds. Little attention has been given to the distinct features of the initial phase of cART and the longterm maintenance phase of viral suppression, once stable stop of viral replication is reached.

However, there are a few fundamental differences in these two phases: During the initial induction phase we face a situation of massive viral turnover within the lymphatic tissue. In an average patient with a blood viral load of 4.5 log_{10} cp/ml, ten billion (10^{10}) new viral particles are created every day(5). As a result of the error prone nature of the viral (reverse) transcriptase (rt) this massive viral replication leads to the creation of a large number of mutations. On average, during the replication of one viral genome the viral rt creates one mutation in a million basepairs (10^{-6}). The length of the viral genome is 9kbp, or roughly 10^4. Since mutations occur at random any single basepair of the viral genome can mutate after a single day. Of course, only the fittest viral genome will continue to establish an infection, but as soon as cART start, a new selection force will lead to the development of resistant viruses. This was the situation of HIV-therapy prior to 1996. However, with the combination of three different compounds it became possible to completely stop the development of resistance. In fact, with optimal antiretroviral management resistant viruses will disappear, as has been recently shown for Switzerland by the Swiss HIV Cohort study(6).

Still, cART with a triple combination has significant drawbacks. Most regimens contain two nucleotides, either abacvavir or tenofovir combined with 3TC (or the very similar combound FTC).

While 3TC has a very long record in HIV therapy with almost 25 years of clinical experience. It is not only a compound with very limited side effects and can be purchased at lower prices as a generic compound, it also has the advantage of excellent penetration in sanctuary sites. The two other compounds (abacavir or tenofovir) both have the disadvantage of significant long-term side effects.

The main reason to combine three compounds to treat HIV infection is to prevent the escape of drug-resistant mutants. But this risk is especially pronounced during the initial phase of therapy when massive viral replication occurs(7). However, the situation is completely different for the phase of HIV maintenance. As mentioned above, viral replication (and also viral evolution) is completely stopped during this phase. Thus, the change of development of mutations resulting in resistant viral particles is markedly reduced.

This difference of the two phases has been clearly shown in many clinical trials. When the first cART was established in 1995, triple cART was compared to an intervention arm with single protease-therapy which failed within a few months. However, in the last decade, a number of studies demonstrated excellent results for 80 to 90% of individuals treated during the maintenance phase with

protease-monotherapy(8). Thus, during the virologically “silent” maintenance phase of HIV-therapy, antiviral suppression appears to be much less demanding. While very strict adherence to cART dosing is crucial for a successful establishment of full viral suppression, patients who fail with their regular drug intake, face much less consequences, i.e. the virus is much more “forgiving”(9).

Protease monotherapy was evaluated in a number of studies worldwide. The major disadvantage of this treatment, quite uniformly shown in all studies, was the increased likelihood of viral “blips”. Viral “blip” refers to the occurrence of a detectable viral load at levels around 50 to 200 HIV-RNA copies/ml which are usually followed by a level below 50 cp/ml. The risk of development of a complete viral failure during protease-monotherapy was low in most studies, but certainly increased compared with triple cART. Two risk factors for viral failure were established in some studies (not consistently, mostly one or the other factor): CD4 Nadir < 200/µl was established as the most important risk factor for failure, followed by the (short) duration of cART prior to treatment switch to monotherapy.

With the extended experience in HIV-monotherapy, another factor was detected that might limit the potency of monotherapy: Poor penetration of protease inhibitors into sanctuaries (such as the brain and the genital tract) was another mechanism leading to viral failure of protease monotherapy as shown in studies evaluating the viral replication in these sanctuaries(10). However, the risk for the development of isolated viral replication in sanctuaries cannot be predicted in an individual. As a consequence, the use of protease monotherapy is now discouraged by most experts in HIV therapy (and also by the guidelines).

As a consequence of these limitations of protease-monotherapies, some groups continued to further develop strategies of bi-therapies with protease inhibitors combined with the nucleotide 3TC(11,12). Although a few studies evaluating this strategy demonstrated an excellent virological suppression with this combination, enthusiasm for this combination was limited due to the significant long-term adverse reaction of boosted protease inhibitors (mainly increase in lipid levels and myocardial infarction).

With the introduction of integrase inhibitors on the market, a new partner for HIV-bi-therapy did appear and several studies started to evaluate new bi- and monotherapies. In fact, the dual combination of Dolutegravir with 3TC resulted in excellent viral load suppression for 32 weeks in an adequately sized study(15).

On the contrary, some centers have started to publish retrospective data on Dolutegravir monotherapy with mixed results. Observational studies of single arm pilot studies or retrospective analyses for about 24 week duration have been presented. Results were -controlled,24-week data have been presented the only integrase inhibitor that can be taken once daily has already been studies in a number of small investigator driven studies. While all these small studies demonstrated continued viral load suppression for up to 24 weeks in the majority of patients, virological failure was nevertheless demonstrated in around 10% of these patients all of these studies. The reason for these failures remains to be investigated. P. Vernazza is a principal investigator of a large randomized study within the Swiss HIV Cohort evaluating the long term value of Dolutegravir monotherapy.

Any reduction in drug exposure for antiretroviral therapy will lower the risk for the development of side effects. In addition, limiting the number of drugs in an antiviral combination will also result in reduced cost. Today, the federal office for public health estimates lifetime costs for cART at about half a Million CHF per infected individual. Therefore a special focus of drug saving strategies should be the evaluation of drugs that are off patent and could be distributed as generic drugs.

One class of drugs for this purpose, would be the non-nuclear RT-inhibitors (NNRTI). While efavirenz is already on the generic market, it’s use has been limited because of frequent neuropsychological side effects. On the other hand, nevirapine (NVP), the first NNRTI that entered the market in the early 90ies has an excellent record for long-term tolerance. Among all the NNRTIs and Protease inhibitors, NVP is the only one that has no effect on lipid levels (if any, lowering cholesterin and triglyceride). The reason, why NVP is not very frequently used by many physician is the relatively frequent (15-20%) occurrence of hypersensitivity reactions (either skin or liver enzyme) with this drug. Since undetected elevation of liver enzyme can result in liver failure during extended treatment with NVP, many physicians did not use this drug, despite its low price.

However, in St. Gallen we have started in 2003 to use this drug for the antiviral maintenance therapy, after it became apparent that the rate of hypersensitivity reactions was much smaller in patients who have already a suppressed viral load. As a consequence, NVP is the most frequently used backbone in St. Gallen with approximately 250 patients on NVP. Within the Swiss cohort, more than 1200 patients are stably treated with a NVP containing regimen.

One outstanding characteristic of NVP is its high penetration into sanctuaries. Among all antiretroviral drugs, NVP is probably the one with the best documented penetration rate in the genital tract and in

the CSF. NVP is the only drug for which high levels in brain tissue have also been shown and the drug is also used with excellent results in HIV-encephalopathy / HIV-dementia.

With the experience we have with protease monotherapy it does not make sense to evaluate NVP as a monotherapy, since the addition of 3TC to NVP has a very limited risk of an added toxicity and the price of this drug is already below 200.- CHF /mt. In addition, the two drugs, still have the potential to be combined in a single pill (400mg NVP, 300mg 3TC, still below the usually cited maximum of 1gr/pill).

Own experience in the field

The ID clinic in St. Gallen was the first clinic worldwide that investigated the use of boosted-Protease-Monotherapy almost 15 years ago in a small study (n=12)(13). The study was the first to demonstrate excellent 48 week suppression during maintenance, but it also already showed the increased rate of viral blips and also an inacceptable rate of side effects under boosted indinavir.

As a consequence, the next study – this time including other centers of the SHCS to include a total of 30 patients - was evaluating boosted the effect of atazanavir monotherapy on sanctuaries (genital tract and CSF)(10) .

This study was the first to demonstrate the limitation of antiretroviral penetration of proteases. The next step was conducted in the MOST study with all SHCS centers, where the CSF penetration was evaluated in a large randomized study with 100 patients on Lopinavir-Monotherapy(14). However, that study had to be terminated early due to the demonstration of unacceptably high viral breaktrough in the CSF.

Currently, Pietro Vernazza is a co-investigator in a SNF-project evaluating dolutegravir monotherapy in approximately 300 patients. However, this strategy has the disadvantage of much higher prize (approx. 800.-/Mt.).

The reasons why we were not able to propose a NVP-based bi-therapy study was the lack of preliminary data that NVP in combination with 3TC could in fact maintain viral suppression in a small number of patients (before the conduction of a large study). Therefore, we propose this small pilot study as a basis for a large randomized study comparing NVP + 3TC with a NVP based standard combination.

3.2 Investigational Product (treatment, device) and Indication

No investigational product is involved in this study. As described in 3.1, the study examines the efficacy of two instead of three standard antiretroviral drugs.

3.3 Preclinical Evidence

n.a.

3.4 Clinical Evidence to Date

See summarized in 3.1

3.5 Dose Rationale / Medical Device: Rationale for the intended purpose in study (pre-market MD)

See 3.1.

3.6 Explanation for choice of comparator (or placebo)

n.a.

3.7 Risks / Benefits

Reducing the drug combination from three to two drugs will not result in an increased risk of adverse events. However, the main concern of a reduction of active compounds in an antiretroviral therapy relates the potential loss of antiretroviral activity. As discussed in the background section, the risk of treatment failure is much lower in the situation of maintenance therapy when the patient has a long-term suppressed viral load.

Loss of activity of a treatment requires the development of viral mutations associated with resistance. However, in order to develop a mutation, the virus must replicate in vivo (in the presence of the selection pressure).

Nevertheless, even if not expected based on theoretical considerations, development of resistance is a major concern. The following strategies to minimize the risk are implemented:

- HIV-RNA testing every 4 weeks in order to rapidly detect every slightest increase in viral replication
- Early communication to all patients of every individual treatment failure within this study
- Strict rules for premature study termination
- A-priori definition of a rescue therapy in case of treatment failure

3.8 Justification of choice of study population

This pilot study was planned as a preparation of a large comparative study to evaluate the bi-therapy with nevirapine and lamivudine within the Swiss HIV Cohort study (SHCS). The sample size (n=10) was defined based on discussions with HIV experts within the SHCS. Most experts agreed that they would be supportive to conduct a large-scale study within the SHCS if 9 of 10 patients would have succeeded with this therapy in a 24 week pilot study (range 8-10 successful patients). Considering the potential risk of treatment failure (see 3.7) the number of patients should also be limited. In addition, a similar number of participants (n=12) was also used in the first PI-monotherapy study (13).

4. AT A FORMAL DISCUSSION OF THIS TRIAL WITHIN THE SCIENTIFIC BOARD OF THE SHCS SOME EXPERTS PROPOSE TO INCREASE THE NUMBER OF PATIENTS FOR THIS PILOT STUDY TO 20. THE NUMBER OF ACCEPTABLE FAILURES AFTER DOUBLING THE SAMPLE SIZE WOULD REMAIN THE SAME, I.E., STOPPING CRITERIA WOULD REMAIN THE SAME (MAXIMUM TWO FAILURES WITHIN THE FIRST 24 WEEKS OF THERAPY IN 20 PATIENTS). STUDY OBJECTIVES

4.1 Overall Objective

The purpose of this study is to demonstrate the potential of NVP + 3TC bi-therapy in a limited number of patients (pilot study).

4.2 Primary Objective

The hypothesis is that at least 18 of 20 patients will maintain viral load suppression <50 copies/ml for at least 24 weeks.

4.3 Secondary Objectives

After the termination of the 24 week study patients may opt to continue the experimental combination therapy as long as viral load suppression is maintained. As a secondary objective the viral failure rate during follow up for 96 weeks will be observed.

4.4 Safety Objectives

No safety endpoint

5. STUDY OUTCOMES

HIV-RNA is measured every 4 weeks during the 24 week study period in blood plasma.

In the occurrence of an HIV-RNA value > 100 copies /ml, the HIV-RNA measurement is repeated with 14 days. The following definitions apply:

- Viral load **suppression** is defined as an HIV-RNA value of <50 cp/ml
- **Viral blips** are defined as HIV-RNA values 50-100 cp/ml or an HIV-RNA value above 100 cp/ml followed by a second measurement below 100cp/ml
- Viral failure is defined as a confirmed HIV-RNA measurement of >100cp/ml

5.1 Primary Outcome

The primary endpoint is the number of patients with no viral failure during the 24 weeks of primary observation period (24 weeks). Successfully suppressed patients may opt to prolong the experimental therapy beyond the primary study endpoint. Regular checks of viral load are requested every 2 months for the first year of bi-therapy and every 3 month beyond that period.

5.2 Secondary Outcomes

- Fraction of patients with no viral failure during the open 96 weeks of secondary observation period.
- Number of patients demonstrating viral blips over the total observation period (fraction of blips per number of VL-measurements) over the total observation period

Other Outcomes of Interest

None

5.3 Safety Outcomes

Other than viral failure, no safety outcome is reported

6. STUDY DESIGN

6.1 General study design and justification of design

Open label, non comparative, single arm pilot study

6.2 Methods of minimising bias

n.a.

6.2.1 Randomisation

n.a.

6.2.2 Blinding procedures

n.a.

6.2.3 Other methods of minimising bias

n.a.

6.3 Unblinding Procedures (Code break)

n.a.

7. STUDY POPULATION

Patients followed in the HIV Clinic at the Kantonsspital St. Gallen fulfilling the eligibility criteria.

7.1 Eligibility criteria

7.1.1 Inclusion criteria

- >18 Years old

- Patients on a stable Nevirapine based HIV triple therapy for at least 6 months
- All viral load measurements (at least 3) in the past 14 months confirmed full suppression (HIV-RNA < 50 cp/ml)
- Duration of viral load suppression (including occasional blips) of more than 2 years
- No previous failure of any NNRTI based therapy
- Availability of a rescue therapy based on an evaluation of the past medical history.

7.1.2 Exclusion criteria

The presence of any one of the following exclusion criteria will lead to exclusion of the participant, for example:

- Chronic hepatitis B infection necessitating treatment with Tenofovir
- Any condition that might interfere with the patients capability to understand the experimental nature of the study

7.2 Recruitment, screening, follow up

Consecutive recruitment of patients visiting the HIV Clinic for their 6-monthly control visit. Before inclusion of patients in the bi-therapy study, a rescue therapy must be defined (s.7.4.1).

At baseline, patients switch their HIV-maintenance to Nevirapine and lamivudine.

7.3 Criteria for withdrawal / discontinuation of participants

7.3.1 Definition of a rescue therapy

Before inclusion in the study, two investigators will have to check based on the individual treatment history of the patient, whether a rescue therapy can be defined in case of viral failure under the experimental therapy. The proposed rescue therapy must be documented in the chart and in the informed consent. Both investigators will have to sign their agreement with the rescue therapy in the patient informed consent. With their signature, the investigators confirm the existence of an alternative HIV therapy in case of treatment viral failure and the selection of the best alternative treatment for the patient.

7.3.2 Procedures in case of viral failure

Any patient who fails the experimental therapy will have to be informed by phone immediately (within 48 hrs). Rescue therapy is established as soon as possible.

7.3.3 Premature study termination due to futility

Since the main goal of the study is to provide sufficient evidence to start a large comparative trial the trial must be stopped prematurely if a certain number of failures (outlined in 11.3) is reached.

In case of futility, i.e. premature study termination, all patients will be reached by phone and informed about the study termination. Patients will be asked to come for a termination visit, bi-therapy stopped and pre-study treatment will be continued (if deemed adequate).

8. STUDY INTERVENTION

8.1 Identity of Investigational Products (treatment / medical device)

8.1.1 Experimental Intervention (treatment / medical device)

Regular prescription drugs are given (Viramune®, 3TC®)

8.1.2 Control Intervention (standard/routine/comparator treatment / medical device)

n.a.

8.1.3 Packaging, Labelling and Supply (re-supply)

n.a.

8.1.4 Storage Conditions

n.a.

8.2 Administration of experimental and control interventions

8.2.1 Experimental Intervention

n.a.

8.2.2 Control Intervention

n.a.

8.3 Dose / Device modifications

Standard dosing is used for the bi-therapy with Viramune and 3TC (400mg / 300mg).

8.4 Compliance with study intervention

Patients are used to excellent adherence. The number of pills per day (2) and dosing (qd.) will be exactly the same as with the treatment before.

8.5 Data Collection and Follow-up for withdrawn participants

Date collection is performed within the patient chart. One single table including all patients (SHCS-ID, Year of birth) with their HIV-RNA dates and values will be generated.

8.6 Trial specific preventive measures

8.6.1 Procedures in case of viral failure

Any patient who fails the experimental therapy will have to be informed by phone immediately (within 48 hrs). Rescue therapy is established as soon as possible.

8.7 Concomitant Interventions (treatments)

Will be collected at each visit according to the standard procedures of the clinical observation of all patients.

8.8 Study Drug / Medical Device Accountability

The monthly drug supply will be given to the patient at each 4-week visit and documented in the electronical data collection system as well as in the patient chart.

8.9 Return or Destruction of Study Drug / Medical Device

n.a.

9. STUDY ASSESSMENTS

HIV-RNA detection at screening and every 4 weeks (week 4 to 24) after initiation treatment change.

9.1 Study flow chart(s) / table of study procedures and assessments

See page 4

9.2 Assessments of outcomes

9.2.1 Assessment of primary outcome

Results of viral load measurement will be checked by one of the principal investigators. Patients will be called in for a repeated testing in the case of an HIV-RNA measurement above 100 cp/ml within 14 days of the increased value. Any HIV-RNA result above 50 cp/ml will be documented in the patient chart with a report of the actions taken.

9.2.2 Assessment of secondary outcomes

For patients who prefer to continue on the bi-therapy after termination of the 24 weeks observation period, 2-monthly HIV-RNA measurement is recommended until week 48 and 3-monthly testing thereafter. Otherwise, assessment is the same as for 9.2.1

9.2.3 Assessment of other outcomes of interest

n.a.

9.2.4 Assessment of safety outcomes

n.a.

9.2.5 Assessments in participants who prematurely stop the study

Participants deciding to stop the study prematurely (not because of viral failure) will be asked to continue on their pre-study antiretroviral regimen.

9.3 Procedures at each visit

Blood draw (8 ml plasma separating tube), detection of HIV-RNA at each visit (4-24 weeks)

10. SAFETY

Any information other than HIV-RNA will only be documented in the source document (patient chart).

10.1 Reporting of serious adverse events (SAE) and other safety related events

All SAEs must be reported immediately and within a maximum of 24 hours to the Sponsor-Investigator of the study. The Sponsor-Investigator will re-evaluate the SAE and return the form to the site.

SAEs resulting in death are reported to the local Ethics Committee (via local Investigator) within 7 days.

Reporting of SUSARs

A SUSAR needs to be reported to the local Ethics Committee (local event via local Investigator) and to Swissmedic for category B and C studies (via Sponsor-Investigator) within 7 days, if the event is fatal, or within 15 days (all other events).

Reporting of Safety Signals

All suspected new risks and relevant new aspects of known adverse reactions that require safety-related measures, i.e. so called safety signals, must be reported to the Sponsor-Investigator within 24 hours. The Sponsor-Investigator must report the safety signals within 7 days to the local Ethics Committee (local event via local Investigator) and to Swissmedic in case of a category B or C study.

11. STATISTICAL METHODS

No statistical analysis will be performed.

11.1 Hypothesis

The purpose of this study is to demonstrate the potential of NVP + 3TC bi-therapy in a limited number of patients (pilot study)

The hypothesis is that at least 18 of 20 patients will maintain viral load suppression <50 copies/ml for at least 24 weeks.

11.2 Determination of Sample Size

No formal sample size calculation possible. Other simplification-therapies (e.g. Dolutegravir Mono) have reported failure rates in the range of 20%. In previous discussions with other SHCS experts (e.g. Alexandra Calmy) experts would generally support the start of this Nevirapine based multicentre study if a pilot study in at least 20 patients would reveal a success rate in 18 of 20 (90%) patients.

11.3 Statistical criteria of termination of trial

In order to limit harm in case the experimental study reaches futility, two safety measures are included

a) Staggered inclusion

After inclusion of the first 5 patients, inclusion will be interrupted until a total number of 10 observation months is reached. The observation period is counted after the return of the laboratory result (HIV-RNA).

b) Premature stopping rules

If one of the following conditions is met, the study will be prematurely terminated:

- More than one virological failure among the first 8 weeks of observation in the first five patients
- More than two failures observed in the first 5 patients
- More than tree failures in anytime during the 24 week observation of up to 20 patients included in the study

If the study is prematurely terminated all successfully treated patients will be switched back to the pre-study antiretroviral therapy.

11.4 Planned Analyses

11.4.1 Datasets to be analysed, analysis populations

See 8.5.

11.4.2 Primary Analysis

Number of treatment failures at week 24

11.4.3 Secondary Analyses

n.a.

11.4.4 Interim analyses

The failure rate in the study is documented on an ongoing manner in order to immediately detect a possible failure of the planned bi-therapy.

One interim analysis will be performed as outlined in 11.3 b).

The final analysis will be done when all patients have completed 24 weeks of follow up (provided the study was not prematurely terminated according to the stopping rules (11.3).

11.4.5 Safety analysis

n.a.

11.4.6 Deviation(s) from the original statistical plan

n.a.

11.5 Handling of missing data and drop-outs

n.a.

12. QUALITY ASSURANCE AND CONTROL

12.1 Data handling and record keeping / archiving

According to existing KSSG SOP

12.1.1 Case Report Forms

All data are documented in the patient chart. Only one excel table will be filled with all HIV-RNA data and patient ID (SHCS-ID).

12.1.2 Specification of source documents

All information is collected in the electronic patient chart of the clinic as the source document.

12.1.3 Record keeping / archiving

All study data must be archived for a minimum of 10 years after study termination or premature termination of the clinical trial.

12.2 Data management

All data entries will be performed by the PI.

12.2.1 Data Management System

Only one form in excel format. Will be documented in the Clinic specific project folder.

12.2.2 Data security, access and back-up

n.a.

12.2.3 Analysis and archiving

n.a.

12.2.4 Electronic and central data validation

n.a.

12.3 Monitoring

n.a.

12.4 Audits and Inspections

n.a.

12.5 Confidentiality, Data Protection

Direct access to source documents will be permitted for purposes of monitoring (12.3), audits and inspections (12.4). Access to the source documents is open to all active collaborators of the infectious diseases clinic.

12.6 Storage of biological material and related health data

n.a.

13. PUBLICATION AND DISSEMINATION POLICY

Publication of the pilot study will be performed regardless of the result of the study. It is planned to submit an abstract for CROI 2018 and publish the result as a letter to the editor in AIDS or a similar journal.

14. FUNDING AND SUPPORT

14.1 Funding

No funding source has yet been sought. Submission to the research funds of the Kantonsspital St. Gallen is planned.

14.2 Other Support

n.a.

15. INSURANCE

Insurance will be provided by the Sponsor. A copy of the electronic certificate is filed in the project folder of the study at the Clinic Infectious diseases and hospital epidemiology.

16. REFERENCES

1. d'Ettorre G, Baroncelli S, Micci L, Ceccarelli G, Andreotti M, Sharma P, u. a. Reconstitution of intestinal CD4 and Th17 T cells in antiretroviral therapy suppressed HIV-infected subjects: implication for residual immune activation from the results of a clinical trial. PloS One. 2014;9(10):e109791.

2. Joos B, Fischer M, Kuster H, Pillai SK, Wong JK, Böni J, u. a. HIV rebounds from latently infected cells, rather than from continuing low-level replication. Proc Natl Acad Sci. 28. Oktober 2008;105(43):16725–30.

3. Gueler A, Moser A, Calmy A, Günthard HF, Bernasconi E, Furrer H, u. a. Life expectancy in HIV-positive persons in Switzerland: matched comparison with general population. AIDS Lond Engl. 7. November 2016;

4. Ryom L, Boesecke C, Gisler V, Manzardo C, Rockstroh JK, Puoti M, u. a. Essentials from the 2015 European AIDS Clinical Society (EACS) guidelines for the treatment of adult HIV-positive persons. HIV Med. Februar 2016;17(2):83–8.

5. Perelson AS, Neumann AU, Markowitz M, Leonard JM, Ho DD. HIV-1 dynamics in vivo: virion clearance rate, infected cell life-span, and viral generation time. Science. 15. März 1996;271(5255):1582–6.

6. Scherrer AU, von Wyl V, Yang W-L, Kouyos RD, Böni J, Yerly S, u. a. Emergence of Acquired HIV-1 Drug Resistance Almost Stopped in Switzerland: A 15-Year Prospective Cohort Analysis. Clin Infect Dis Off Publ Infect Dis Soc Am. 15. Mai 2016;62(10):1310–7.

7. Scherrer AU, von Wyl V, Böni J, Yerly S, Klimkait T, Bürgisser P, u. a. Viral suppression rates in salvage treatment with raltegravir improved with the administration of genotypic partially active or inactive nucleoside/tide reverse transcriptase inhibitors. J Acquir Immune Defic Syndr 1999. 1. Mai 2011;57(1):24–31.

8. Bierman WFW, van Agtmael MA, Nijhuis M, Danner SA, Boucher CAB. HIV monotherapy with ritonavir-boosted protease inhibitors: a systematic review. AIDS Lond Engl. 28. Januar 2009;23(3):279–91.

9. Shuter J. Forgiveness of non-adherence to HIV-1 antiretroviral therapy. J Antimicrob Chemother. April 2008;61(4):769–73.

10. Vernazza P, Daneel S, Schiffer V, Decosterd L, Fierz W, Klimkait T, u. a. The role of compartment penetration in PI-monotherapy: the Atazanavir-Ritonavir Monomaintenance (ATARITMO) Trial. AIDS Lond Engl. 19. Juni 2007;21(10):1309–15.

11. Crespo M, Navarro J, Martinez-Rebollar M, Podzamczer D, Domingo P, Mallolas J, u. a. Improvement of BMD after Switching from Lopinavir/R Plus Two Nucleos(T)ide Reverse Transcriptase Inhibitors to Lopinavir/R Plus Lamivudine: OLE-LIP Substudy. HIV Clin Trials. Mai 2016;17(3):89–95.

12. Arribas JR, Girard P-M, Landman R, Pich J, Mallolas J, Martínez-Rebollar M, u. a. Dual treatment with lopinavir-ritonavir plus lamivudine versus triple treatment with lopinavir-ritonavir plus lamivudine or emtricitabine and a second nucleos(t)ide reverse transcriptase inhibitor for maintenance of HIV-1 viral suppression (OLE): a randomised, open-label, non-inferiority trial. Lancet Infect Dis. Juli 2015;15(7):785–92.

13. Kahlert C, Hupfer M, Wagels T, Bueche D, Fierz W, Walker UA, u. a. Ritonavir boosted indinavir treatment as a simplified maintenance „mono"-therapy for HIV infection. AIDS Lond Engl. 9. April 2004;18(6):955–7.

14. Gutmann C, Cusini A, Günthard HF, Fux C, Hirschel B, Decosterd L-A, u. a. Randomized controlled study demonstrating failure of LPV/r monotherapy in HIV: the role of compartment and CD4-nadir. AIDS Lond Engl. 24. September 2010;24(15):2347–54.

15. Baril JG, Angel JB, Gill MJ, Gathe J, Cahn P, van Wyk J,Walmsley S: Dual Therapy Treatment Strategies for the Management of Patients Infected with HIV: A Systematic Review of Current Evidence in ARV-Naive or ARV-Experienced, Virologically Suppressed Patients. PLOSone September 2016;11(2):e0148231.

17. APPENDICES

n.a.
