# Supplementary material for: Novel dual HIV maintenance therapy with nevirapine plus lamivudine retain viral suppression through 144 weeks—A proof-of-concept study
Source: PLoS One. 2020 Sep 23;15(9):e0237770. doi: 10.1371/journal.pone.0237770 (PMC7511013; doi:10.1371/journal.pone.0237770)
Supplement: Attachment — Submitted filename: 20191202_response-to-reviewers.pdf [file pone.0237770.s003.pdf]

Response (bold) to REVIEWER COMMENTS (italic)

Reviewer #1
1) Evidently this is an interesting piece of work that requires further evaluation in a larger study for patients who are currently taking nevirapine. The paper states that nevirapine is a safe drug to switch to "regardless of CD4 count" in those who are undetectable on ART. I think that there are some significant questions about nevirapine as a stable switch strategy which are not addressed in this paper. The evidence concerning this question is observational and retrospective. The comparator in these papers is rate of HSR in anti-retroviral naive patients with low CD4 counts starting nevirapine not people starting ART containing alternative agents. Indeed in the paper of Wit et al, 2008 for those patient switching to nevirapine in the "high-high" group, the lower limit of the confidence interval for the odds of HSR only just touches 1. This is a drug assoicated with liver toxicity and HSR but with guidelines which limit the risk of these adverse events. I think it is important to clarify this as, while the ambition to maintain use of older, cost-effective drugs is admirable, readers should be aware of this limitation in the context of the wide availability of other third agents and strategies in the current era. If we are only comparing nevirapine with nevirapine then this strategy has limited scope.

We thank the reviewer for these constructive and helpful comments. Absolutely, the caution of using Nevirapine must remain clearly articulated. We have adapted the introduction accordingly. We specified that the evidence is based on cohort studies and changed the sentence from “ART can be safely switched” to “the risk of HSR after switching to a NVP-based regimen is lower” (Line 76). At the same time, we would like to emphasize that we do not propose a switch in our manuscript, but instead propose dual therapy (DT) with NVP and 3TC as maintenance for patients who tolerate NVP more than 6 months.

2) I think you might want to just check through the references again - line 181 indicates reference 8 with respect to reduced HSR, but this reference is about CNS PK.

We totally agree and thank the reviewer for this comment. The reference was changed from reference 8 to references 10,12 and 13.

Reviewer #2
Thank you for this interesting and, as you say in your conclusion, surprising study. I think this deserves publication but with some amendments:

1) Abstract conclusion: "Our findings are surprising but do not falsify the null-hypothesis that dual therapy is inferior to standard therapy" - I do not understand this statement. Do you mean to say that "this pilot study, though promising, is not powered to prove whether this therapy option is non-inferior to standard of care" – please revise so clear

We thank the reviewer for all these valuable comments and suggestions. In this sentence we want to clearly express that the results do not support the use of this DT maintenance therapy due to the small sample size. However, they are surprising and do support the conduct of a properly sized clinical trial. To elucidate this intention, both the summary and the last paragraph of the manuscript have been adapted.

2) You abstract conclusion point "having the advantage of reduced adverse events and cost" should be removed. You have not demonstrated, as far as I can see, reduced AE in this small, single arm pilot and in the era of largely generic NRTI there may be no cost advantage - indeed, in England, the difference between 3TC and TDX/FTC generic is less than £1 a week....

You are right, a study design without a control group cannot prove a reduction of adverse events. Therefore, we removed this sentence from the abstract conclusion. However, it is evident that the risk for cART-associated long-term side effects is reduced with a DT versus triple cART. Concerning reduced costs, this of course varies by country. In Switzerland, we currently still do not have generic TDF/FTC that's why the difference with 3TC is still significant with about £140 a week. With regard to NVP, the cost saving potential is even more than 50% compared to a therapy with e.g. dolutegravir.

3) I think to describe your own findings as 'surprising' you need to better justify your rationale for undertaking the trial and expand on the information provided to participants with respect to risk of failure or resistance development

Thank you for this comment. Because there is no previous experience with NVP-containing DT and in order to rapidly detect a possibly increased risk of viral failure, special safety precautions have been included in the protocol. First, a stopping rule was implemented (stop of the study in the event of viral failure in two patients) and second, the protocol required a stepwise inclusion (3 steps). This is additionally illustrated in the study flow (*Figure 1*). In order to further clarify this issue, we modified the sentence (Line 90-93), adding "in particular to prevent the development of resistance in the event of virus failure". All patients included signed informed consent containing information on the potential risk of failure and development of resistance and this information was approved by the local ethical review board.

4) The fact that NVP is not recommended in any high-income country guidelines should be acknowledged; similarly you need to provide some clinical context for recommending a regimen like this - why choose NVP/3TC over more established tripe/dual options?

Thank you, that's correct. In order to make this clear, we have both changed the introduction (Line 61-64) and the conclusion (Line 205-206). Justification of this regimen is covered in the introduction as well as in the conclusion (more than 20 years of experience with documented long-term tolerance and efficacy over decades, compartment penetration, prize).

5) Please include some discussion as to the value of pilots e.g. PADDLE is an example of promising pilot findings being replicated in a larger single-arm study and then large RCTs, PI/r + MVC is an example whereby promising single-arm results were not replicated in RCTs

You're right, a successful pilot does not guarantee success in a large RCT. To disclose this concern, we have added an additional point to the limitations (Line 191-193).
