# Supplementary material for: Novel dual HIV maintenance therapy with nevirapine plus lamivudine retain viral suppression through 144 weeks—A proof-of-concept study
Source: PLoS One. 2020 Sep 23;15(9):e0237770. doi: 10.1371/journal.pone.0237770 (PMC7511013; doi:10.1371/journal.pone.0237770)
Supplement: Attachment — Submitted filename: 20200729_nvp3tc_p2p_reply_r2_submitted.pdf [file pone.0237770.s004.pdf]

Response (**bold**) to REVIEWER COMMENTS (*italic*)

Main points

1. What is the definition of 'fully suppressed' viral load (inclusion criteria of trial)? <1 copy/mL? <=50 copies/mL? The actual breakdown of the viral load distribution at week 0 could be shown in Table 1

We agree and thank the reviewer for this comment. A “fully suppressed” viral load was defined by HIV-RNA ≤ 50 copies/mL. We added this information under “Methods”. Since all 20 participants had undetectable HIV-pVL at the beginning of the study, a respective sentence was added to the results.

2. Are there stored samples available in which to measure low-level drug resistance? It is currently possible to measure resistance even when viral load is very low. If authors could show that there was, say, no 103N or 184V in minor populations at week 96 this finding would decrease concerns around clinical use of this combination. HSR and cost are less important issues in my opinion. Most mono/dual combinations with low resistance barrier have shown inferiority when compared to standard triple regimens in head to head RCTs. The lack of resistance data should at least be mentioned in the Discussion.

We do agree with the reviewer’s comment regarding the use of resistance test in clinical trials. This pilot study was designed to rule out a high risk of treatment failure of this dual regiment, before the conduct of a properly powered non-inferiority trial which is currently running. Throughout the manuscript, we always maintain this argument. However, following the valid suggestion of reviewer #3 to assess possible low-level resistance, we attempted - in collaboration with Prof. Thomas Klimkait from the Department of Biomedicine, Molecular Virology, University of Basel Switzerland - next-generation sequencing (NGS) enrichment of the stored sample with elevated HIV viral load of 55 copies/mL. The procedure was as follows. Centrifugation to concentrate the virus from 1mL, RNA was converted to cDNA and amplified using a validated nested PCR protocol (inner reaction simultaneously used 2 primer pairs to increase the sensitivity of amplification from clinical specimens. DNA was run on a 1% gel. Controls were as expected, but we could not obtain a suitable band from the specimen. Nevertheless, the gel was cut in the expected size range with a haze of a signal (figure available upon request) and gel-extracted using the Macherey Nagel extraction kit. The product was sent to Dr. Martin Däumer, SeqIT, Kaiserslautern, Germany, for next-generation sequencing (MiSeq). Unfortunately, no analyzable product was obtained (<50 reads, which did not pass quality control). The group at SeqIT is VERY experienced with HIV resistance testing by NGS, and according to them, the finding hints an insufficient copy number of virus in the sample. Of course, we cannot rule out that the quality of this specimen with an original viral load of 55 copies went down due to various manipulations until the final testing. Although this diagnostic took several weeks, partly due to the COVID-19 pandemic, we can unfortunately neither confirm nor exclude low-level resistance and have therefore added this as a limitation in the discussion. Nevertheless, we thank the reviewer for this suggestion.

3. With a sample size of 20 patients it is easy to show spaghetti plots with the full viral load trajectories for the all study population, including the 96 week before and after the inclusion into the pilot maintenance study. It is mentioned on line 190-192 that However, in this population, all patients kept full HIV pVL suppression and even the frequency of blips with DT was equal to the pre-study period where patients received cART with 3 compounds. This is not supported by data shown in Figure 2 because this includes average proportions and it is not possible to say whether blips where more/less frequent over the pilot study than over the previous period in individual patients. Indeed, there seems to be evidence in aggregate of people moving from the ≤1 copy/mL stratum (78% vs 86%) to the 2-20 copies/mL stratum (19% vs 10%) under dual therapy. Spaghetti plots will give more insights and possibility to develop specific statistical tests to compare the frequency of blips before and after the switch.

We would like to thank the reviewer for this commentary and made a spaghetti plot (Figure 3a) of the results. Furthermore, a dot plot was done individually for each patient and combined (Figure 3b/c). We agree with the reviewer that - although the total number of blips is very low - equality is not visible. We have therefore revised the statement in the manuscript (lines 190-192) accordingly and suggest replacing the previous Figure 2 with the new Figure 3c in the manuscript to improve visualization of the results.

4. There is no control group in this trial so the only possible comparison is with historical values of viral load measured over the previous period under triple therapy. Intermittent time series approach for average viral load levels comparing the slope before and after the switch to dual can be used to confirm that there is no evidence for a difference in the slopes in the two periods. This auto-regressive method is very powerful and commonly used in mono-arm trials with no control group now also in HIV research.

We thank the reviewer for this suggestion. In collaboration with the statistician of the local clinical trials unit (PD Dr. Sabine Güsewell), we tried to implement intermittent time series models and interrupted time series models to assess differences of viral blips pre-study and post-study period. However, as many patients experienced 0-1 blip only, unfortunately these models could not be calculated meaningfully. In order to provide calculated comparison of blips in both periods, we compared the proportion of measurements with detectable HIV-RNA or HIV-RNA ≥ 20 copies/ml before and during the study. The results suggest no difference in both periods and were added to the manuscript.

5. Legend for Figure 2. I would modify the label for the stratum '>50' into '50-100' copies/mL. Or where there people who showed blip >100 copies/mL?

As reported in the manuscript, the maximal single HIV pVL was 55 copies/mL. We absolutely support the suggestion to modify the label for the stratum '>50', however we suggest to replace Figure 2 by Figure 3c.

Other Points

1. Line 44-46. Afterwards, viral replication is completely suppressed and a simplified treatment with two antiretroviral compounds (dual therapy – DT) or even one substance (monotherapy) may control viral replication on the long run. This sentence needs to be qualified. None of the mono-therapy regimens have shown particularly encouraging results; same story applies to some of the dual combinations, shown to be inferior to triple therapy (e.g. those including maraviroc etc).

We agree, that there is little evidence for this statement, this is why we state „*may* control…" and explain in the following sentence the underlying hypothesis. Indeed, when indinavir was first evaluated in the nineties, starting patients on monotherapy did result in failure as it was seen with other substances too. However, we were able to show continued viral load suppression in monotherapy maintenance for selected patients (Kahlert, 2016, PMID 26661659). We did rephrase the sentence to clarify that this is a hypothesis. The ongoing, properly sized trial is currently testing this hypothesis.

2. Line 171. Typo NVP and 3TC, not und

Thank you, this typo has been corrected.

3. A previous reviewer pointed out that sentence in lines 233-234 was not particularly clear. This has now been revised in the current version in 'In summary, given the sample size, our findings do not reject the null-hypothesis that dual therapy is equally effective as standard therapy'. I think that this is still inaccurate; the study does not support equivalence of the dual therapy with triple therapy because there is no control group. Lack of a plausible causal argument is more important than statistical power.

We fully support the notion that the study does not support equivalence of the DT. That's why it is stated "our findings do not reject the null-hypothesis that dual therapy is equally effective as standard therapy".

4. It is mentioned in the Conclusions (lines 237-238) and Abstract that 'A properly sized multicentre non-inferiority trial is ongoing to further evaluate the value of this DT maintenance strategy'. Does this trial really exist? There seems to be no sign of such a trial in https://clinicaltrials.gov/. It would be useful to give more information on the status of such trial.

Indeed, the study has been approved in 2019 and is registered in the Swiss National Clinical Trials Portal (https://www.kofam.ch/en/snctp-portal/searching-for-a-clinical-trial/, Identifier SNCTP000003395). The study has been started in 4 centers in Switzerland is running and currently more than 100 patients reached 48 weeks. We will provide information on the results as soon as possible.
